# Neuropsychological Symptoms among Workers Exposed to Toluene and Xylene in Two Paint Manufacturing Factories in Eastern Thailand

**DOI:** 10.1155/2015/183728

**Published:** 2015-07-28

**Authors:** Anamai Thetkathuek, Wanlop Jaidee, Sastri Saowakhontha, Wiwat Ekburanawat

**Affiliations:** ^1^Department of Industrial Hygiene and Safety, Faculty of Public Health, Burapha University, Chonburi 20131, Thailand; ^2^Department of Public Health Foundations, Faculty of Public Health, Burapha University, Chonburi 20131, Thailand; ^3^Department of Community Medicine, Family Medicine and Occupation Medicine, Faculty of Medicine, Burapha University, Chonburi 20131, Thailand; ^4^Occupational Medicine Center, Samitivej Sriracha Hospital, Chonburi 20230, Thailand

## Abstract

The study analyzed the exposure factors that may lead to neuropsychological symptoms among 92 workers who were exposed to xylene and toluene and 100 workers who were not exposed to the solvents. The airborne concentration of xylene and toluene was evaluated with personal passive badges. The levels of methyl hippuric acid and hippuric acid in urine were assessed, and interviews were performed to observe the neuropsychological symptoms that may result from exposure to the solvents. The result showed that the average concentration for the exposed group of xylene in the paint company working environment was 2.7 (SD = 2.4) ppm and the average concentration of toluene was 9.5 (SD = 10.4) ppm. The average level of methyl hippuric acid in urine was 78 (SD = 74.7) mg/g creatinine. Factors that affected the neuropsychological symptoms included the following. (1) *The impact of age*: the risk (*adjusted odds ratio*) for getting psychosomatic symptoms in persons over 40 and exposed to xylene was 9.5 and the aOR of those exposed to toluene was 8.3. (2) *The impact of not providing personal protective equipment* was found to be sleep disturbance; it was found that the aOR of those exposed to xylene was 3.9, and the aOR of those exposed to toluene was 4.4. In summary, periodic examination of workers by occupational physician is needed for detection of early neuropsychological effects, especially psychosomatic symptoms, and sleep disturbances.

## 1. Introduction

Manufacturers in developing countries have widely used solvents as raw material in different productions, such as plastic, coating, paint, and glue [1–3]. Although nowadays water-based paints are preferred, rather than solvent-based paints [4], solvents are still used in the manufacture of specialty paints. In Thailand, xylene and toluene are used as major components in combination with other solvents in the paint production process. These toxic aromatic hydrocarbons may be dispersed during the production process and affect the health of exposed factory workers [5].

Xylene and Toluene can be absorbed into the blood via three routes including the lungs, the skin, and gastrointestinal tract. Xylene and toluene, especially, can be deposited in adipose tissue [6]. Absorbed xylene and toluene are metabolized by mixed-function oxidase enzyme systems into methyl hippuric acid, o-cresal (for xylene), and benzoic and hippuric acid (for toluene) before being excreted in the urine. Among the metabolites, methyl hippuric acid, o-cresal, and hippuric acid are a biomarker in the biological monitoring of occupational exposure to xylene and toluene [7]. Methyl hippuric acid and hippuric acid are widely accepted indicators in the assessment of xylene and toluene in mixed solvent exposed workers in Thailand. The previous studies report a good correlation of measurements of exposure to airborne xylene and toluene with their metabolites in urine [8].

Exposure to xylene and toluene can cause acute and chronic effects in human body systems. Workers exposed to these substances may experience neuropsychological symptoms [9–11], for example, mild headache, fatigue [12], and cognitive disorders. The impacts of the solvents on mental conditions and emotional disorders [13, 14] may include depression, headache, fatigue, anxiety, drunken feelings, and insomnia [15]. Other disorders may include difficulty in concentrating or remembering [16], fatigue, sleepiness, and clumsiness.

There are many factors in the use of solvents associated with neurological disorders, including personal factors, such as age and gender and inappropriate behaviors such as alcohol use and smoking, not wearing personal protective equipment such as a mask and gloves [12], inappropriate working conditions [9], long working hours, and also channels of exposure to the body such as the respiratory tract and the skin.

The exposure assessment allows the biological monitoring of workers by assessing their exposure to substances from the working environment, for example, a personal sampling for the toluene concentration [17], and an assessment of the environmental concentrations of the solvents that can enter the body via the respiratory tract and skin. The assessment of chemicals can be based on parent compounds or metabolites, such as from blood and urine [9]. The assessment of chemicals in urine is preferred rather than a blood test because it is a noninvasive method. The relationship between the level of toluene in urine and the concentration of toluene in the working environment as found by using the personal passive badge has already been demonstrated [17].

There are various methods for assessing the long term impacts on health, for example, epidemiology [18], and questionnaires, namely, Q16 [19], Q18 [20], and Euroquest (EQ) [21], which can be applied for screening and the diagnosis for the toxicity of solvents. The Euroquest questionnaire has been tested for sensitivity and specificity and can be applied for screening and diagnosis for toxicity of the solvents [22].

Despite the results concerning exposure to solvents and the impact of exposure on the neuropsychological symptoms of workers [12, 16], most studies in Thailand assessed the exposure to high concentrations of solvents in the working environment [23]. More evidence is required regarding the exposure to low concentrations of xylene and toluene and regarding the risk factors for neuropsychological symptoms. Therefore, the authors' intentions are to examine the factors affecting the disorders and neuropsychological symptoms of workers exposed to low levels of xylene and toluene in paint manufacturing. This will help to set guidelines for the health screening and for the development of policy to take care of workers at risk.

## 2. Research Methodology

The methodology of the present study was analytical research. The cross-sectional data was obtained from workers in two paint manufacturing facilities. The data collection period was from April 2011 to June 2011.

## 3. Population and Sample

The population included workers in paint manufacturing that used solvents in combination with xylene and toluene. The researcher coordinated with all five paint manufacturers in the Eastern Region; however, responses were obtained from executives of two manufacturers to participate in the research. The determination of the sample size applied a sample-size formula that would allow use of multiple logistic regressions to find the factors that had an impact on health [24], and the calculation of the sample size resulted in 173 persons. The characteristics of the process in paint production are similar; thus the cluster sampling for two plants was selected for this study. Every worker who worked in a process related to solvent exposure was eligible as a study subject.

The researcher collected the data from all 92 volunteer workers exposed to solvents in the production process (exposure group) and selected 100 workers from a frozen food factory as a control group (nonexposure group) by randomly sampling worker's names from a list of the human resources department. For the exposed group, the number of workers in the plant #1 and #2 was 58 workers and 34 workers, respectively.

A nonexposed group of workers from food frozen manufacturing was selected as a control group because solvents are not used in the production process.

The inclusion criteria for the exposure group were an age between 20 and 60 years; employment in the production process; an exposure to the solvents for at least 3 months; and voluntary participation in the research. All workers chosen for the study were not compelled. They signed consent forms to participate. The present research was approved by the Ethical Review Committee of Burapha University.

## 4. Instruments and Materials Used in the Research

The research instruments consisted of four types:* (1) interview forms; (2) air sampling tools; (3) gas chromatography; and (4) high performance liquid chromatography (HPLC)*. First, the researcher instructed and explained the objectives, techniques, methods, process, and procedure of research to the research assistant to ensure consistency in data collection. After that, the researcher explained the research objectives and necessary information to the volunteers and asked them to sign consent forms to participate in the research.

The* interview form* consisted of three parts.* Part 1* (general information): this part includes age, gender, education, income, smoking history (nonsmoker, ex-smoker, and smoker), and alcohol use (nondrinker, ex-drinker, and drinker).* Part 2* (working history): this part includes exposure to solvents including working hours; characteristics of the solvent-related job categorized by operational practice, namely, low exposure, moderate exposure, and high exposure; and the use of personal protective equipment (Table 2).* Part 3* (illness history): this part of interview form was created from a literature review and consisted of 20 questions. The scoring contained two levels: no symptom (value = 0 point) and having a symptom (value = 1 point). The researcher and team collected data by interviewing the workers directly. The interview takes about 5–10 minutes per worker. The neuropsychological disorders were classified into six groups [9] (1) neurological symptoms: facial paresthesia, weakness, peripheral neuropathy, anosmia, hyposmia, change of taste, dizziness, and numbness, (2) psychosomatic symptoms: headache, sweating with unknown cause, dyspnea, palpitation, lethargy, fatigue, loss of libido, nausea, vomiting, and loss of appetite, (3) mood symptoms: restlessness and depression, (4) memory and concentration: difficulty in concentration, (5) tiredness: drowsiness, and (6) sleep disturbances: insomnia and difficulty in sleeping.

An* interview form* used for data collection was prepared. The researcher sent the newly designed interview form to experts to consider and verify its structural validity, content validity, comprehensiveness, and language appropriateness. The experts included one physician specializing in occupational medicine and two professors specializing in occupational health and safety. After all the experts had considered the form, the researcher revised the interview form as recommended.

An* air sampling tool* was used to collect air samples by industrial hygienist in order to measure the concentration of xylene and toluene in the working environment. Air samples containing xylene and toluene were collected by using a passive badge sampler (3M 3500 Organic Vapor Monitor). Personal air sampling was conducted for eight hours starting from the installation of the passive badge on the collar of the workers before beginning work on their shift until the end of their shift. This air sampling was conducted in the middle of the week. After that, the passive badge was immediately placed on ice in a foam-box. Then the passive badges were transferred and stored in a refrigerator at the temperature −20°C in the laboratory of the Faculty of Public Health at Burapha University.

The passive badges were sent for analysis to the laboratory of the Bureau of Occupational and Environmental Diseases of the Ministry of Health. Time Weight Average (TWA) was determined and used in statistical analysis. A gas chromatography machine was used for analysis. For the gas chromatography, a Column Aqua-Wax Polyethylene Glycol was used to analyze the toluene and xylene concentrations of the passive badges. Carbon disulfide was used for extraction. The calibration and quality control was carried out by following the NIOSH method 1501 [25]. The standard threshold limit value (TLV) of xylene in working environment by ACGIH [26] is 100 ppm, and the standard TLV of ACGIH [26] of toluene is 20 ppm, respectively.

For the* urine collection*, at least 10 mL per person of urine was collected after eight hours at the work station. Urine samples were collected in the end of shift at the end of workweek. The samples were immediately placed in an ice box for transfer to a −20°C freezer in the laboratory of the Faculty of Public Health of Burapha University until being carried to the laboratory in Bangkok for analysis.

High performance liquid chromatography (HPLC) and spectrophotometry (Star Dust MC 15) were used to analyze the metabolite concentration of xylene and toluene, namely, methyl hippuric acid (mg/gram creatinine) and hippuric acid (mg/gram creatinine) in the urine of the workers exposed to the solvents, at the Occupational and Environmental Department of the Ministry of Public Health in Bangkok. The calibration and quality control was performed as per the NIOSH method 8301, fourth edition [27]. The standard biological exposure indices (BEI) of methyl hippuric acid in the urine are 1.5 g/g creatinine [28], and BEI of hippuric acid in the urine are 1.6 g/g creatinine [26].

## 5. Data Analysis

This study analyzed the statistical information using the software SPSS/PC (Statistical analysis for The Social Science/Personal Computer). The general information of the working history, exposure to the solvents, the disorders, and neurological and psychological health effects are tabulated, along with means, standard deviation, median, minimum, and maximum.

A comparison of the concentration of xylene and toluene in the working environment, and of methyl hippuric acid and hippuric acid, between the nonexposed group (factory 1) and the exposed group (factories 2 and 3) was done using ANOVA as statistical test.

An analysis of the factors that had an impact on the neurological and psychological health effects was done using multiple logistic regression,* odds ratio (OR)*, and confidence interval at 95% CI. The independent variables included gender, age, alcohol use, work duration (years), the concentration of xylene or toluene in working environment (parts per million, ppm), and the use of personal protective equipment, respectively. In this analysis, the gender ratio of exposure and nonexposure groups is different; thus adjustments were done to control for certain variables in logistic regression.

## 6. Results

### 6.1. Demographic Characteristics

The study found the following demographic characteristics. Of the 192 subjects, there were 92 workers exposed to the solvents (exposed group) and 100 workers not exposed to the solvents (nonexposed group). Most of the workers exposed to the solvents were males (83 employees, 90.2%); most had an average age of 33.1 years (SD = 7.5) years; most of them (52 persons, 56.5%) graduated from high school; and 65 persons (70.7%) were married. Concerning their smoking history, it was found that most of the workers (60 persons, 65.2%) were nonsmokers, and that most of those who smoked had been smoking for approximately 12.6 years (10.2%). Concerning their alcohol use, it was found that most of the workers (70 persons, 76.1%) were currently drinking alcohol (Table 1).

### 6.2. Work History

Concerning the work duration of the employees, it was shown that most of them (48 persons, 52.1%) had been working for more than five years. Their average years of work duration were 7.7 (SD = 6.1). Most workers (82 persons, 89.1%) worked for eight hours per day. In each week, most workers (69 persons, 75.0%) worked for six days per week. Concerning overtime work, it was found that many workers (40 persons, 43.5%) worked overtime for more than 10 hours per week. Their average overtime working hours were 15.5 (SD = 8.5) hours.

### 6.3. The Analysis of the Airborne Xylene and Toluene Concentration

The analysis of the airborne xylene and toluene concentration for the exposed group indicates that workers exposed to xylene had a mean exposure of 2.7 parts per million (SD = 2.4, Median 1.9 (Min, Max: 0.1, 13.1)). The mean toluene exposure was 9.5 parts per million, (SD = 10.4, Median 4.9 (Min, Max: 0.5, 48.7)). When statistically comparing the airborne concentration of xylene and toluene between the exposed group and the nonexposed group, it was found that the concentration of xylene and toluene was significantly different (*p* value < 0.001).

### 6.4. The Concentration of Methyl Hippuric Acid and Hippuric Acid

The concentration of methyl hippuric acid had a mean of 78.3 mg/gram creatinine (SD at 74.7, Median 50.8 (Min, Max: 0.3, 331.7) mg/gram creatinine). When statistically comparing the concentration of methyl hippuric acid between the exposed group and the nonexposed group, it was found that the concentration of methyl hippuric acid in the urine was not different (*p* value > 0.05) (Table 3).

The concentration of hippuric had* mean* value of 575.23 mg/gram creatinine (SD = 566.9 and Median was 423.51 (Min, Max: 5.5, 2626.9) mg/gram creatinine). When statistically comparing the concentration of hippuric acid between the exposed group and the nonexposed group, it was found that the concentration of hippuric in the urine was statistically different (*p* value < 0.001) (Table 3).

This study found a correlation between airborne xylene and urinary methyl hippuric acid levels (*p* < 0.001) and found a correlation between airborne toluene and urinary hippuric acid levels (*p* < 0.001).

### 6.5. The Impact on the Neurological and Psychological Symptoms

Regarding the current symptoms of the workers, it was found that they had demonstrable neurological and psychological symptoms. Their psychosomatic symptoms included* headache, sweating with unknown cause, dyspnea, palpitation, lethargy, fatigue, loss of libido, nausea, vomiting, and loss of appetite* (65 persons, 70.7%). Concerning their neurological symptoms, most workers had* facial paresthesia, weakness, peripheral neuropathy, anosmia, hyposmia, change of taste, dizziness, and numbness *(52 persons, 56.5%) (Table 4).

### 6.6. The Risk Factors of the Neurological and Psychological Symptoms

The analysis showed that age, concentration of xylene, and the use of personal protective equipment had an influence on the neuropsychological symptoms. The details are explained below.

The factor that influenced psychosomatic symptoms* (headache, sweating with unknown cause dyspnea, palpitation, lethargy, fatigue, loss of libido, nausea, vomiting, and loss of appetite)* was the age of the workers exposed to xylene and toluene. It was found that those workers aged over 40 years, who were exposed to xylene, had* adjusted odds ratio (aOR)* of 9.5 for these symptoms (95% CI 13.2–68.8). In case of those who were exposed to toluene, there was an* OR *of 8.3 for this symptom group (95% CI 1.2–58.5).

Another factor that affected* psychosomatic symptoms* was* work duration*. It was found that work duration of more than 5 years being exposed to xylene had aOR of 0.2 (95% CI 0.02–0.9). Those who worked more than 5 years and who were exposed to toluene had aOR of 0.16 (95% CI 0.02–0.9). In addition, it was shown that work duration more than 5 years had an impact on mood symptom among those who were exposed to toluene with aOR at 0.1 (95% CI was 0.02–0.9).

The factor that affected sleep disturbance was wearing personal protective equipment. It was shown that not wearing personal protective equipment had an impact on sleep disturbance. Those who were exposed to xylene while not wearing personal protective equipment had aOR for sleep disturbance at 3.9 (95% CI 1.1–13.8). Additionally, those who were exposed to toluene while not wearing personal protective equipment had an aOR of 4.4 (95% CI 0.2–15.8).

The factor that affected* tiredness* was the concentration of toluene. It was found that the range of toluene concentration in the working environment at 15.23–30.33 parts per million corresponded with lessening of this symptom: aOR of 0.2 (95% CI 0.03–0.9) (Table 5).

## 7. Discussion

The limitations of the present study included the short employment terms of the workers, which did not allow the observation of the impact of long term exposure to xylene and toluene. Moreover, the interviews about the disorders due to xylene and toluene were based on the perceptions of the study's subjects. They were not diagnosed by a medical doctor. Thus, it was not possible to accurately assess actual disorders due to xylene and toluene. Also, the assessment of neurological and psychological disorders was based on a literature review and all questions were grouped according to Mandiracioglu et al. [9], which may not bring all the questions that may indicate effects of exposure. Also, because the number of male workers in the nonexposed and the exposed group is different the researchers adjusted for gender in the Logistic Regression Model. Data analysis indicated that sex is not associated with various symptoms. In this present study, the majority of the exposed group was male (90.2%), and 76.7 of them were currently drinking alcohol 2 or 3 times per week; however, we assume that this relatively low frequency of alcohol intake may not affect any neuropsychological symptoms.

The present study assessed the concentration of xylene and toluene in the working environment of paint manufacturers. It was found that the concentration of xylene and toluene was below the TLV [26]. The TWA of airborne xylene levels was <100 ppm, and toluene was 20 ppm [26]. The current study found that the urinary hippuric acid levels in the exposed group were higher than in the nonexposed group, but that was not the case for methyl hippuric acid. However, among paint workers in the current study, 6.78% of paint workers had hippuric acid >1.6 g/g creatinine [28], the ACGIH-recommended biological exposure index for hippuric acid in urine. In this present study, workers who had urinary hippuric acid higher than the BEI were working in jobs with high exposure potential, such as grinding pigment, tank cleaner, paint sprayer, and filling paint into containers.

Among the paint workers, the median methyl hippuric acid level and median hippuric acid level were 50.75 mg/g creatinine (range, 0.3–331.7) and 423.5 mg/g creatinine (range, 5.5–2626.9), respectively. This study found a correlation between airborne xylene and urinary methyl hippuric acid levels (*p* < 0.001) and found a correlation between airborne toluene and urinary hippuric acid levels (*p* < 0.001), respectively.

The concentration of xylene measured here was higher than that in the study of Ukai et al. [29]; however, the concentration was lower than that in the study of Chen et al. [30] and Mao et al. [31], respectively. Concerning the concentration of toluene in the working environment, it was shown that the concentration was higher than that in the study of Hopf et al. [32].

Concerning the assessment of the toluene concentration in the urine in the hippuric acid form, some have argued that the result might vary due to metabolism. It has also been reported that the toluene concentration in urine was a more sensitive biomarker than hippuric acid [33]; however, many studies have found an association between metabolites in the urine and the concentration of toluene [33, 34]. Therefore, it was widely used as a toluene-exposure biomarker [35, 36].

In fact, ACGIH [26] recommended using O-cresal in urine as biomarker for exposure to toluene, instead of using hippuric acid as a biomarker of exposure. In Thailand, however, there have been limitations regarding analytic technologies, thus hippuric acid has been more favored as a biomarker of exposure. Most importantly, no food with a preserving agent should be taken before the test (benzoic acid) [37].

Concerning the analysis of the factors, namely, gender, age, alcohol use, work duration (years), the concentration of xylene in the working environment (parts per million), and the wearing of personal protective equipment, all of which may affect neurological and psychological disorders, it was found that the age of the workers had an impact on the psychosomatic symptoms, consisting of* headache, sweating with unknown cause, dyspnea, palpitation, lethargy, fatigue, loss of libido, nausea, vomiting, and loss of appetite*. This study found that those in the age group over 40 years who were exposed to xylene and toluene were at risk of having psychosomatic symptoms. This may be due to the interaction between the exposure to the solvents and the age [38].

In a similar study by Condray et al. [13], the psychiatric symptoms of painters who had a chronic exposure to low levels of organic solvents were reported. The painters showed a subclinical pattern of personality dysfunction, which was measurable at the age above 25 years. However, neurological and mental disorders have been related to many factors, and researchers have not been able to completely control all variables. Therefore, the result from the present study suggests that older age poses higher risk of such disorders, and that safety officer should pay more attention to the health of workers in the age range above 40 years. The current study implies that workers who were exposed to xylene and toluene were not affected by the neurological symptoms, psychosomatic symptoms, mood symptoms, memory and concentrating, and sleep disturbance. This may be due to a low concentration, as our median of xylene exposure level was 1.99 ppm (range, 0.09–13.1) ppm, and toluene exposure was 4.9 ppm (range, 0.5–48.7).

The present study did find that the toluene concentration in the working environment was a factor related inversely to tiredness. However, the relationship between toluene concentration and tiredness by crude logistic regression was insignificant; thus, the tiredness may come from other cofactors.

The finding was in contrast to the study of Klaucke et al. [39], who stated that people felt* headache, nausea, dizziness, and vomiting* after an exposure to xylene at a concentration of 700 parts per million for an hour. On the other hand, the study of Alfaro-Rodríguez et al. [40] found that toluene exposure at a concentration of 1–80 parts per million (15 parts per million on the average) caused disorders, such as,* headache, nausea, and vomiting*. This was opposed to a laboratory test in which volunteers were immediately exposed to toluene [40]. Our study results are not consistent with the study of Condray et al. [13], who reported psychiatric symptoms, such as anxiety and moodiness, among people with a chronic exposure to low levels of organic solvents [14].

The current findings did not correspond to the studies of many researchers, who stated that the exposure to mixed solvents caused neurophysiological and psychological disorders [41–44]. However, additional assessments should be performed regarding the type of solvent and the severity of exposure. Also, in the present study, the number of workers continuously exposed to xylene and toluene was insufficient since the average work duration (years) of the workers was only 6.9 (6.0) years.

Moreover, concerning the wearing of personal protective equipment in those exposed to xylene and toluene, it was found that workers who did not wear personal protective equipment were at risk of* sleep disturbance*. Therefore, personal protective equipment was able to prevent the exposure to xylene and toluene in the working environment that may have entered the body via breathing or skin during working hours.

In this present study, the researcher noticed that the workers usually wore the chemical mask when working on a hazardous task for a short time, such as mixing chemicals; then they took it off. It is suggested that the manufacturer should have strict control of the working environment. If control of the working environment is inadequate, the chemicals will spread into working environment, and the workers will be exposed to the chemicals which are accumulated in the body, resulting in adverse medical conditions.

A surprising result of this present study was that exposure to xylene or toluene had a lower impact on* psychosomatic symptoms* for those with work duration greater than 5 years than the exposure of workers who worked less than one year. However when analyzing the relationship by the crude regression, it was shown that duration of the work was not associated with psychosomatic symptoms.

This study results' were in contrast with the study of Chen et al. [30], who found that the duration of exposure to the solvents was extremely associated with the lifetime cumulative exposure to the solvents. In the present study, this result may be because the body is not capable of adapting itself to the exposure in the short term, compared to the exposure in the long term. Alternatively, the inverse association of time on the job with identification of psychosomatic symptom may be the result of other variables. However, neurotoxicity due to lead (Pb) exposure in this present study was not necessary because lead has been removed from the paint production process.

In summary, being over 40 years of age is a factor affecting psychosomatic symptoms among workers exposed to xylene or exposed to toluene. Additionally, not wearing personal protective equipment, for example, respirator, is a factor affecting sleep disturbance symptoms among both xylene and toluene exposed groups. However, low concentrations of xylene and toluene have been not related to any symptoms except the concentration of toluene which is associated with reduced tiredness symptom.

The results of the study suggest that primary prevention of occupational exposure to mixed solvents especially xylene and toluene is very important, although the workers are working under low concentration of these kinds of solvent. The manufacturers should measure the concentration of xylene and toluene in the working environment as regulated by the law, including the airborne sampling with personal passive badges. Officials should also regularly assess the exposure of xylene and toluene to the body's blood or urine samples from workers at risk.

Health effects from exposure to low concentration of xylene and toluene among workers should be monitored, which include a plan to monitor the health screening for neuropsychological, especially psychosomatic, symptoms and sleep disturbances by having the safety personnel or hygienists use a standardized interview form with workers and pay close attention to workers over 40 years of age, particularly those who have been working for more than 5 years; and provide education about personal hygiene to workers who work with solvents to prevent adverse impacts on their health.

Moreover, periodic physical examination of workers by an occupational physician as regulated by law is needed for detection of early neuropsychological effects. If abnormalities are found in any employee, according to the* regulation and procedures for health examination in employees, which was announced since 2004* by the Ministry of Labor in Thailand, these employees should be treated immediately and the cause of abnormalities determined immediately.

## Figures and Tables

**Table 1:** Subject characteristics.

Factors	Nonexposed *n* = 100 (52.1%)	Exposed *n* = 92 (47.9%)
Sex		
Male	12 (24.5)	83 (90.2)
Female	37 (75.5)	9 (9.8)
Age (yr)		
Mean (SD) years	33.51 (9.06)	33.14 (7.5)
Median (Min, Max) years	31.0 (20, 53)	31 (22, 53)
Drinking history		
Nondrinker	31 (63.3)	12 (13.0)
Ex-drinker	4 (8.2)	10 (10.9)
Current drinker	14 (28.6)	70 (76.1)
Smoking history		
Never smoker	40 (81.6)	60 (65.2)
Ex-smoker	2 (4.1)	12 (13.0)
Current smoker	7 (14.3)	20 (21.7)

**Table 2:** Work history.

Factors	Nonexposed *n* = 100 (52.1%)	Exposed *n* = 92 (47.9%)	Exposed group (*n* = 92)			
			Xylene exposed group		Toluene exposed group	
			≤2.71 ppm	>2.71 ppm	≤9.52 ppm	>9.52 ppm
			*n* = 56 (60.9%)	*n* = 36 (39.1%)	*n* = 61 (66.3%)	*n* = 31 (33.7%)
Work duration (yr)						
<1	24 (24)	7 (7.6)	2 (3.6)	5 (13.9)	3 (4.9)	4 (12.9)
1–5	38 (38)	37 (40.2)	18 (32.1)	19 (52.8)	26 (42.6)	11 (35.5)
>5	38 (38)	48 (52.1)	36 (64.3)	12 (33.3)	32 (52.5)	16 (51.6)
Mean (SD)	*4.7 (4.3) *	*7.73 (6.1) *	*9.5 (6.8) *	*4.9 (3.5) *	*8.67 (6.9) *	*5.87 (3.9) *
Median (Min, max)	*3 (0, 21) *	*6 (0, 25) *	*6.50 (0, 25) *	*4.0 (1, 15) *	*6.0 (0, 25) *	* 6 (0, 15) *

Characteristics of the solvent related job categorized by operational practice						
Low	0 (0)	30 (32.6)	25 (44.6)	5 (13.9)	30 (49.2)	0 (0)
Moderate	0 (0)	16 (17.4)	11 (19.6)	5 (13.9)	11 (18.0)	5 (16.1)
High	0 (0)	46 (50.0)	20 (35.7)	26 (72.2)	20 (32.8)	26 (83.9)

**Table 3:** Level of xylene and toluene exposure in work environment (ppm) and urinary metabolite (mg/gram creatinine) in non-exposed and exposed group.

	Nonexposed group				Exposed group			
	Xylene	Toluene	Methyl hippuric acid	Hippuric acid	Xylene	Toluene	Methyl hippuric acid	Hippuric acid
	*n* = 49 (nondetectable = 51%)	*n* = 49 (nondetectable = 51%)	*n* = 29 (nondetectable = 71%)	*n* = 66 (nondetectable = 34%)	*n* = 92	*n* = 92	*n* = 73 (nondetectable = 19%)	*n* = 59 (nondetectable = 33%)
Means	0.3	0.5	51.2	202.2	2.7	9.5	78.3	575.2
Standard deviation	0.0	0.8	72.5	188.7	2.4	10.4	74.7	566.9
Median	0.3	0.3	30.3	166.5	1.9	4.9	50.8	423.5
Minimum	0.3	0.1	4.7	1.0	0.1	0.5	0.3	5.5
Maximum	0.4	5.1	297.6	1066.1	13.1	48.7	331.7	2626.9

**Table 4:** Health effects on neuropsychological symptoms.

Neuropsychological symptoms	Nonexposed *n* = 100 (52.1%)	Exposed *n* = 92 (47.9%)	Exposed group (*n* = 92)			
			Xylene concentration		Toluene concentration	
			≤2.71 ppm	>2.71 ppm	≤9.52 ppm	>9.52 ppm
			*n* = 56 (60.9%)	*n* = 36 (39.1%)	*n* = 61 (66.3%)	*n* = 31 (33.7%)
(1) Neurological symptoms	29 (59.2)	52 (56.5)	32 (57.1)	20 (55.6)	37 (60.7)	15 (48.4)
(2) Psychosomatic symptoms	33 (67.3)	65 (70.7)	40 (71.4)	25 (69.4)	47 (77.0)	18 (58.1)
(3) Mood symptoms	14 (28.6)	21 (22.8)	13 (23.2)	8 (22.2)	15 (24.6)	6 (19.4)
(4) Memory and concentrating	18 (36.7)	30 (32.6)	21 (37.5)	9 (25.0)	21 (34.4)	9 (29.0)
(5) Tiredness	16 (32.7)	35 (38.0)	23 (41.1)	12 (33.3)	27 (44.3)	8 (25.8)
(6) Sleep disturbances	15 (30.6)	43 (46.7)	25 (44.6)	18 (50.0)	29 (47.5)	14 (45.2)

(1) *Neurological symptoms*: facial paresthesia, weakness, peripheral neuropathy, anosmia, hyposmia, change of taste, dizziness, and numbness.

(2) *Psychosomatic symptoms*: headache, sweating with unknown cause, dyspnea, palpitation, lethargy, fatigue, loss of libido, nausea, vomiting, loss of appetite.

(3) *Mood symptoms*: being restless, depression.

(4) *Memory and concentrating:* difficulty in concentration.

(5) *Tiredness:* drowsiness.

(6) *Sleep disturbances*: insomnia, difficulty to sleep.

**Table 5:** Factors affecting neuropsychological symptoms among workers exposed to xylene and toluene.

Variables	Xylene exposed group		Variables	Toluene exposed group			
	Psychosomatic symptoms	Sleep disturbances		Psychosomatic symptoms	Mood symptoms	Tiredness	Sleep disturbances
	aOR (95% CI)	aOR (95% CI)		aOR (95% CI)	aOR (95% CI)	aOR (95% CI)	aOR (95% CI)
Gender			Gender				
Female	0.3 (0.1–1.1)	1.37 (0.4–4.9)	Female	0.4 (0.1–1.3)	1.17 (0.3–4.7)	0.9 (0.3–3.5)	1.5 (0.4–5.6)
Age			Age				
25–29	2.4 (0.4–14.2)	3.3 (0.5–20.3)	25–29	2.18 (0.4–13.1)	4.14 (0.4–41.3)	1.9 (0.3–12.7)	3.8 (0.6–22.8)
30–34	3.4 (0.6–19.6)	0.9 (0.2–5.7)	30–34	2.8 (0.5–16.5)	3.4 (0.4–28.7)	0.9 (0.2–5.6)	1.1 (0.2–6.3)
35–39	5.47 (0.8–36.7)	2.9 (0.5–18.6)	35–39	4.2 (0.6–27.5)	6.1 (0.7–57.0)	1.3 (0.2–8.6)	2.5 (0.4–15.3)
>40	9.5 (13.2–68.8)	1.7 (0.3–11.9)	>40	8.3 (1.2–58.5)	3.1 (0.3–31.3)	0.7 (0.1–5.1)	1.4 (0.2–9.2)
Drinking history			Drinking history				
Ex-drinker	—	1.1 (0.20–5.6)	Ex-drinker	0 (0.0)	0.6 (0.10–4.2)	1.4 (0.2–7.7)	0.9 (0.2–5.4)
Current drinker	0.9 (0.3–3.0)	1.4 (0.4–4.9)	Current drinker	1.0 (0.3–3.3)	0.5 (0.1–1.9)	1.4 (0.4–4.9)	1.5 (0.4–5.2)
Work duration (yr)			Work duration (yr)				
1–5	0.8 (0.1–4.1)	1.2 (0.2–6.6)	1–5	0.7 (0.1–3.8)	0.3 (0.1–1.6)	1.01 (0.2–5.5)	0.9 (0.2–4.4)
>5	0.2 (0.02–0.9)	1.04 (0.2–6.7)	>5	0.1 (0.02–0.9)	0.1 (0.02–0.9)	0.9 (0.2–5.9)	0.7 (0.1–4.0)
Xylene (ppm)			Toluene (ppm)				
0.33–6.66	0.5 (0.2–1.8)	2.4 (0.7–7.9)	15.2–30.3	0.3 (0.1–1.2)	0.3 (0.05–1.9)	0.2 (0.03–0.9)	0.7 (0.2–2.3)
>6.66	0.3 (0.04–2.4)	2.51 (0.4–16.4)	>30.33	1.07 (0.1–12.5)	1.20 (0.1–13.5)	4.6 (0.4–49.5)	—
Personal protective equipment			Personal protective				
No	2.1 (0.6–7.2)	3.9 (1.1–13.8)	No	2.2 (0.6–7.8)	1.6 (0.4–5.9)	1.8 (0.5–6.2)	4.4 (1.2–15.8)

*Note*. (1) The reference group for each factor was gender (male); age (19–24 yrs); drinking history (never drink); work duration (<1 yr); xylene (<0.3 ppm); personal protective equipment (yes).

(2) The reference group for each factor was gender (male); age (19–24 yrs); drinking history (never drink); work duration (<1 yr); toluene (<15.23 ppm); personal protective equipment (yes).
